# Experimental data from an open-source DC motor control educational kit for calibration, system identification, and controller validation

**DOI:** 10.1016/j.dib.2026.113274

**Published:** 2026-09-17

**Authors:** Alejandro Von Chong, Dorindo Cárdenas

**Affiliations:** a School of Electrical Engineering, Universidad Tecnológica de Panamá, Víctor Levi Sasso Campus, Panama City, Panama; b Sistema Nacional de Investigación-SENACYT, Panama City, Panama

**Keywords:** System identification, Embedded firmware, PID tuning, Sampled systems

## Abstract

This data article presents raw and processed experimental data collected with the OpenMCT DC motor control educational kit. The dataset includes current-sense calibration data, static motor characterization data, system-identification logs at four sampling times, continuous PI controller validation at four loop periods, discrete controller validation for three controller designs, and an additional chirp-excitation characterization run. The raw acquisition files were exported from the OpenMCT Python/Qt graphical user interface as comma-separated text files containing reference commands, measured speed, loop interval, raw and filtered current-sense values, PWM command, and synchronized digital multimeter measurements. MATLAB scripts, processed files, model tables, screenshots, and exported figures are provided to reproduce the data organization and plots. The records can be used to prepare laboratory exercises in which students identify a motor model and compare its predicted response with experimental measurements. They also provide reference data for evaluating alternative identification and controller-validation methods.

Specifications Table

**Table:** 

Subject	Engineering & Materials science
Specific subject area	Educational tools and open-source alternatives to existing infrastructure: Open-source control education, system identification, and digital control of DC motors
Type of data	Raw text logs, CSV tables, PNG/PDF/FIG figures, screenshots, MATLAB scripts and MAT files
Data collection	Data were collected with the OpenMCT DC motor educational kit described in the related HardwareX article. The setup used a Teensy 4.0 microcontroller, a DRV8874 H-bridge motor driver, a TSINY TS-25GA370H-20 brushed DC motor with encoder feedback, a 12 V supply, the OpenMCT Python/Qt GUI, and a Siglent SDM3045X digital multimeter for current measurements. The dataset contains raw and processed data from the educational kit of the related research article, including DC motor calibration, open-loop characterization, system identification, and continuous/discrete time controller-validation experiments.
Data source location	Institution: Universidad Tecnológica de Panamá City/Town/Region: Panama, Panama Country: Panama
Data accessibility	Repository name: Mendeley Data Data identification number: 10.17632/5xvg43r9r8.2 Direct URL to data: https://data.mendeley.com/datasets/5xvg43r9r8/2
Related research article	A. Von Chong, S. Vargas, J.-F. Duhé, D. Cárdenas, OpenMCT: an open-source DC motor control educational kit, HardwareX 26 (2026) e00794. https://doi.org/10.1016/J.OHX.2026.E00794.

## 1 Value of the data

• The dataset documents a complete experimental control workflow on the same open-source DC motor platform, covering current calibration, static characterization, amplitude-modulated pseudo-random binary sequence (APRBS)-based system identification, continuous PI validation, discrete controller validation, and chirp excitation. The data allow users to follow the progression from raw hardware measurements to model estimation, controller implementation, and experimental response validation.
• The raw logs retain the PWM command alongside measured speed, so users can examine the input actually applied to the motor when interpreting its response. The current-sense records also include DMM sample identifiers and timestamps, allowing users to reconstruct which measurements were paired during calibration.
• Students can use the three model structures fitted to each APRBS acquisition to examine whether adding a pole or zero improves the description of the measured response. Comparing RMSE with AIC and BIC introduces the trade-off between fitting error and model parameter count using experimental data from the same acquisition.
• The PI validation records document the plant model used for tuning and the controller gains deployed at each loop period. The 5 ms controller uses a plant identified from a 2 ms acquisition, providing a teaching example in which the identification sampling period differs from the controller execution period.
• The three discrete-controller cases allow students to reconstruct the implemented difference equations from the archived coefficients. The two integrator cases illustrate a change in gain, while the third introduces a real zero. Their measured responses provide experimental examples for discussing how these controller choices affect closed-loop behaviour.
• The processing scripts allow users to reproduce the published plots from the raw logs without operating the motor. Because the intermediate results are also available, users can modify a processing step and compare its output with the archived result.

## 2 Background

OpenMCT is an open-source educational platform for DC motor control that combines a TSINY TS-25GA370H-20 brushed DC motor with encoder feedback, a DRV8874 H-bridge driver, a Teensy 4.0 microcontroller, and a Python/Qt graphical user interface for real-time visualization, data logging, parameter configuration, system identification, and controller deployment. The related HardwareX article describes the hardware, firmware, graphical interface, design files, bill of materials, assembly procedure, and validation workflow [1].

This dataset was compiled to provide the raw and processed experimental records generated while using the OpenMCT DC motor module for calibration, characterization, system identification, and controller-validation exercises. It extends the hardware description by making the acquisition logs, processing scripts, model-result tables, and exported figures available as reusable data files. The dataset is organized so that each experiment folder contains its raw log, metadata, analysis code, result files, and generated figures [2].

## 3 Data Description

The dataset contains raw and processed data from DC motor calibration, open-loop characterization, system identification, and controller-validation experiments. The files are organized by experiment. Raw acquisition logs are plain-text, comma-separated records generated by the OpenMCT graphical user interface (GUI). The GUI was built using SciPy and the Python Control Systems Library [3,4].

The first two lines store acquisition metadata (Mode and Date), the third line stores variable names, and the following lines store numeric samples. MATLAB scripts reproduce the processing steps and generate the accompanying figures and result files. The raw log columns are REF (reference command), MEAS (measured motor speed in RPM), DT_ms (measured sample interval in milliseconds), CURRENT_RAW (raw current-sense ADC count), CURRENT_AVG (firmware-filtered current estimate), PWM (applied PWM command), DMM_CURRENT_A (digital multimeter current measurement in amperes, when available), DMM_TIME_s (digital multimeter timestamp), DMM_AGE_ms (age of the multimeter sample when the GUI row was written), and DMM_SAMPLE_ID (multimeter sample identifier). Repeated DMM_SAMPLE_ID values refer to the same asynchronous multimeter reading. In the current-calibration acquisition, CURRENT_AVG was calculated using the provisional calibration available during recording. The final calibration was obtained from CURRENT_RAW and DMM_CURRENT_A. Files named *_results.mat store variables produced by the analysis scripts. Files with .png, .pdf, and .fig extensions are exported figures from MATLAB or screenshots from the GUI (Tables 1,2).

**Table 1:** Dataset organization by experimental workflow stage, showing the GUI mode or input signal, nominal sampling time, main reusable files, and repository folder for each acquisition.

Workflow stage	GUI mode / input	Nominal Sampling time	Main reusable files	Folder
Current-sense calibration	System Identification / manual braking	20 ms	`current_calibration.m', `current_calibration_results.mat', exported calibration figures	`01_current_calibration/raw_data.txt'
Identification of static nonlinearities	System Identification / triangular command	20 ms	`static_characterization.m', `static_characterization_results.mat', exported static-map figures	`02_static_characterization/raw_triangular_data.txt'
System identification	System Identification / automatic step-reference sequence	2 ms	GUI model screenshots, `system_identification_model_results.csv', `system_identification_summary.m`	`03_system_identification/02 ms sampling time/raw_data.txt'
		10 ms		`03_system_identification/10 ms sampling time/raw_data.txt'
		20 ms		`03_system_identification/20 ms sampling time/raw_data.txt'
		50 ms		`03_system_identification/50 ms sampling time/raw_data.txt'
Continuous PI validation	Speed Control / APRBS	5 ms	`PID_validation.m', plant/tuning/deployment screenshots, validation figures	`04_continuous_PID_validation/5_ms/raw_data_5ms.txt'
		10 ms		`04_continuous_PID_validation/10_ms/raw_data_10ms.txt'
		20 ms		`04_continuous_PID_validation/20_ms/raw_data_20ms.txt'
		50 ms		`04_continuous_PID_validation/50_ms/raw_data_50ms.txt'
Discrete controller validation	Speed Control / APRBS	20 ms	`Discrete_validation.m', discrete plant/design screenshots, validation figures	`05_discrete_controller_validation/Case_a_integrator_overdamped_gain_of_3.86/raw_data.txt'
		20 ms		`05_discrete_controller_validation/Case_b_integrator_underdamped_gain_of_13/raw_data.txt'
		20 ms		`05_discrete_controller_validation/Case_c_integrator_with_added_zero_at_0.66_gain_of_9.9/raw_data.txt'
Additional chirp characterization	System Identification / Chirp signal	20 ms	`chirp_characterization.m', `chirp_characterization_results.mat', exported chirp figures	`optional_characterization/raw_chirp_data.txt'

**Table 2:** Hardware, measuring instruments and software used for dataset generation.

Component	Model or software	Role in data collection
Microcontroller	Teensy 4.0	Embedded acquisition loop, reference generation, encoder processing, current-sense reading, PWM actuation, and controller execution.
Motor driver	DRV8874 H-bridge	DC motor actuation and IPROPI current-sense output.
DC motor	TSINY TS-25GA370H-20	Brushed DC motor with encoder feedback; 12 V, 300 RPM, 240 pulses per revolution.
Power supply	GA-1202,000	12 V, 2 A motor supply.
Digital multimeter	Siglent SDM3045X	External DC current measurement for current calibration and synchronized current records.
GUI	OpenMCT Python/Qt interface	Serial communication, real-time plotting, data logging, excitation setup, plant identification, controller tuning, and deployment.
Firmware environment	PlatformIO `teensy40', Arduino framework	Firmware build and deployment environment.
Data processing	MATLAB R2024b	Processing scripts, MAT result files, and exported figures.

DATA_DESCRIPTION.md provides the complete file inventory, while DATASET_METADATA.md records the acquisition hardware and software environment. The current_calibration_age_sensitivity CSV and MAT files contain the results obtained by varying the maximum accepted DMM sample age. The continuous_PI_controller_parameters.csv and discrete_controller_parameters.csv files store the controller parameters numerically, and these parameters are also included in the corresponding validation MAT files.

## 4 Experimental Design, Materials and Methods

The GUI and firmware are archived in Firmware/DC_Motor under commit ebdb5643aa06eeb82041b7ba49c13afab9ca68f8. The acquisitions used Python 3.13.5, and the processing scripts were run in MATLAB R2024b Update 6. Package versions and firmware build dependencies are documented in DATASET_METADATA.md. Transfer function simulations in MATLAB require Control System Toolbox (version 24.2 was used in this work).

SDM3045X measurements were read through the PyVISA connection integrated into the OpenMCT GUI. The multimeter was configured for DC current on the fixed 10 A range, with autorange disabled, NPLC = 0.3 (Fast), immediate trigger, and one sample per acquisition [5,6]. The fixed 10 A range was necessary to avoid losing data during the multimeter’s current range switching, mainly due to transient current spikes when energizing the motor. The GUI exported each acquisition as a text log and saved the firmware loop interval in `DT_ms', allowing the time vector to be reconstructed from the data file. The loop interval is derived in the firmware from a microsecond-based timer. Consequently, the archived DT_ms field has a resolution of 0.001 ms, except for the chirp signal test, which has a time resolution of 1 ms. Mean, sample standard deviation, minimum, and maximum DT_ms values were calculated for all 14 acquisitions and are reported in Table 3. Every stored interval equalled the corresponding nominal period. The complete numerical summary is provided in optional_characterization/sampling_time_statistics.csv and sampling_time_statistics_results.mat.

**Table 3:** Statistics of the archived DT_ms field for all acquisitions. SD denotes the sample standard deviation calculated with N − 1 normalization. The stored resolution is 0.001 ms for all acquisitions, except for the chirp acquisition which is 1 ms.

Acquisition (nominal period)	Mean (ms)	SD (ms)	Min (ms)	Max (ms)
Current calibration (20 ms)	20	0	20	20
Static characterization (20 ms)	20	0	20	20
System identification (2 ms)	2	0	2	2
System identification (10 ms)	10	0	10	10
System identification (20 ms)	20	0	20	20
System identification (50 ms)	50	0	50	50
Continuous PI (5 ms)	5	0	5	5
Continuous PI (10 ms)	10	0	10	10
Continuous PI (20 ms)	20	0	20	20
Continuous PI (50 ms)	50	0	50	50
Discrete A (20 ms)	20	0	20	20
Discrete B (20 ms)	20	0	20	20
Discrete C (20 ms)	20	0	20	20
Chirp (20 ms)	20	0	20	20

For current calibration, the GUI recorded the firmware current-sense ADC output while the SDM3045X recorded current asynchronously. The raw log stores the DMM sample value, timestamp, age, and sample identifier for each GUI row. Candidate calibration pairs were restricted to samples with DMM_AGE_ms ≤ 10 ms, and the freshest GUI row associated with each DMM_SAMPLE_ID was retained. An initial linear fit was then calculated between CURRENT_RAW and DMM current in milliamperes. Residual outliers were rejected when their deviation from the median residual exceeded 4*1.4826*MAD, where MAD is the median absolute deviation of the initial-fit residuals. The final calibration was refitted using the retained samples. For the selected 10 ms threshold, 5059 candidate pairs were identified, 141 were rejected by the residual criterion, and 4918 samples were used for the final fit. DMM current was converted to milliamperes using its absolute value because the IPROPI output represents current magnitude. The final fit had an RMSE of 7.108 mA. This value describes the discrepancy between the retained calibration pairs and the fitted line; it does not represent the uncertainty of the multimeter. Fig. 1 shows the calibration fit and the comparison with the DMM measurements.

**Fig. 1:**
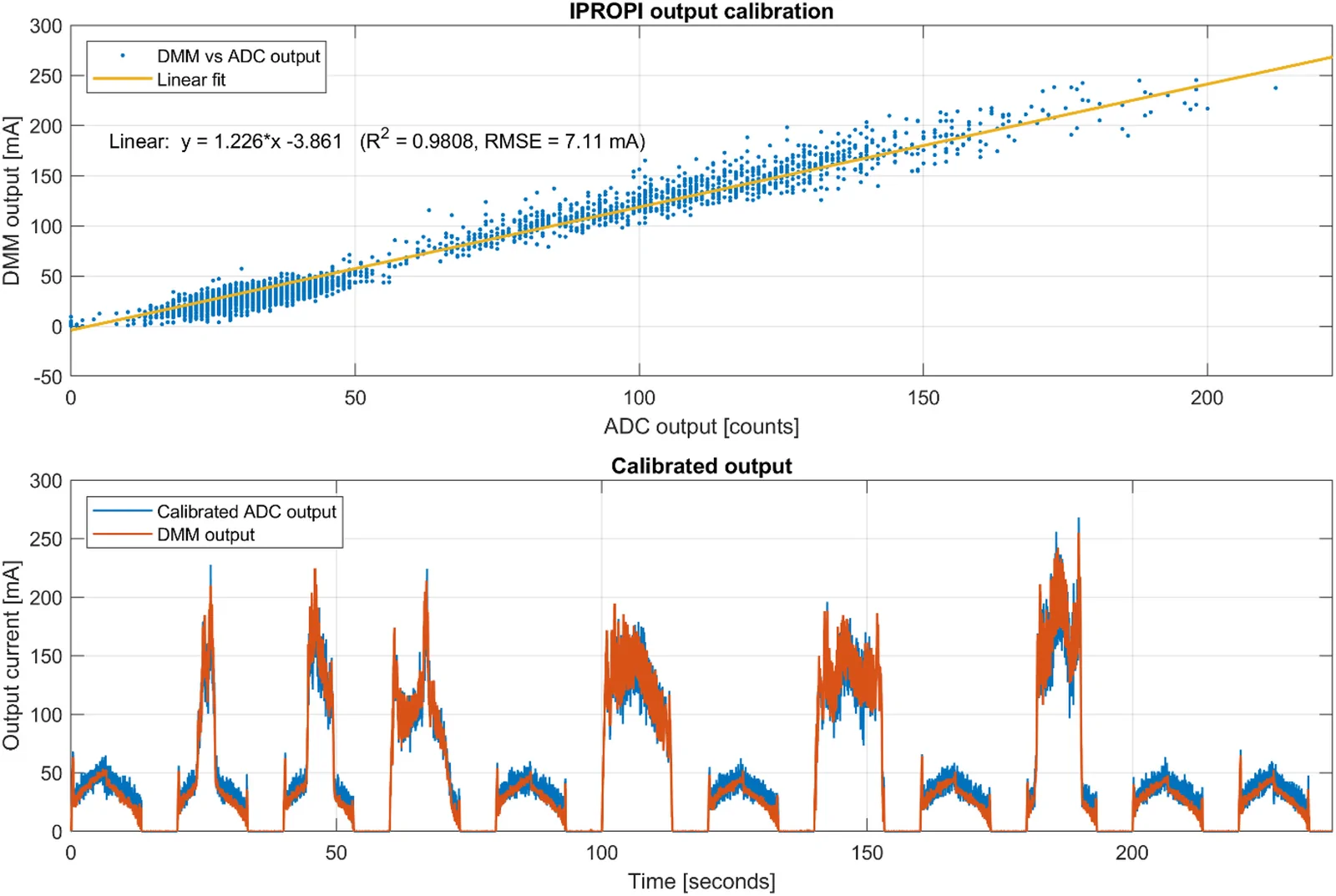
Current-sense calibration data. Top: digital multimeter current as a function of the DRV8874 IPROPI ADC output and fitted linear conversion. Bottom: time-domain comparison between calibrated ADC current and synchronized SDM3045X current samples.

Sensitivity to the DMM synchronization threshold was assessed by repeating the complete calibration procedure for maximum DMM sample ages of 2.5, 5, 7.5, 10, 12.5, 15, and 20 ms while keeping the residual-rejection rule unchanged. Table 4 summarizes this test.

**Table 4:** Sensitivity of the current-sense calibration to the maximum accepted DMM sample age. The complete calibration procedure, including the residual-based outlier rejection stage, was repeated for each threshold. Note that RMSE values are calculated from the retained samples at each threshold; therefore, the number and composition of samples differ between thresholds.

Maximum DMM age (ms)	Total / retained candidate pairs	Rejected (%)	Final slope (mA/count)	Intercept (mA)	RMSE (mA)
2.5	804/777	3.36	1.203	−3.572	6.353
5	2309/2229	3.46	1.220	−3.566	6.703
7.5	3639/3528	3.05	1.225	−3.867	6.970
**10**	**5059/4918**	**2.79**	**1.226**	**−3.861**	**7.108**
12.5	6301/6123	2.82	1.220	−3.788	7.185
15	7247/7023	3.09	1.221	−3.777	7.164
20	9083/8817	2.93	1.222	−3.827	7.269

For static characterization, the motor was driven with a triangular PWM command from 0 to 254 counts at a nominal 20 ms sample time. The processing script reconstructs elapsed time from `DT_ms', separates ascending and descending sweep branches, bins measured speed by integer PWM command, and stores the resulting arrays and figures. The triangular command had a 40 s period. The dead-zone estimate required three consecutive PWM-bin means at or above 15 RPM. The linear fit used equally weighted bin means from 30 to 220 PWM counts and had an RMSE of 0.436 RPM. No statistical outlier rejection was applied. Fig. 2 shows the triangular-sweep measurements and the resulting static input–output map.

**Fig. 2:**
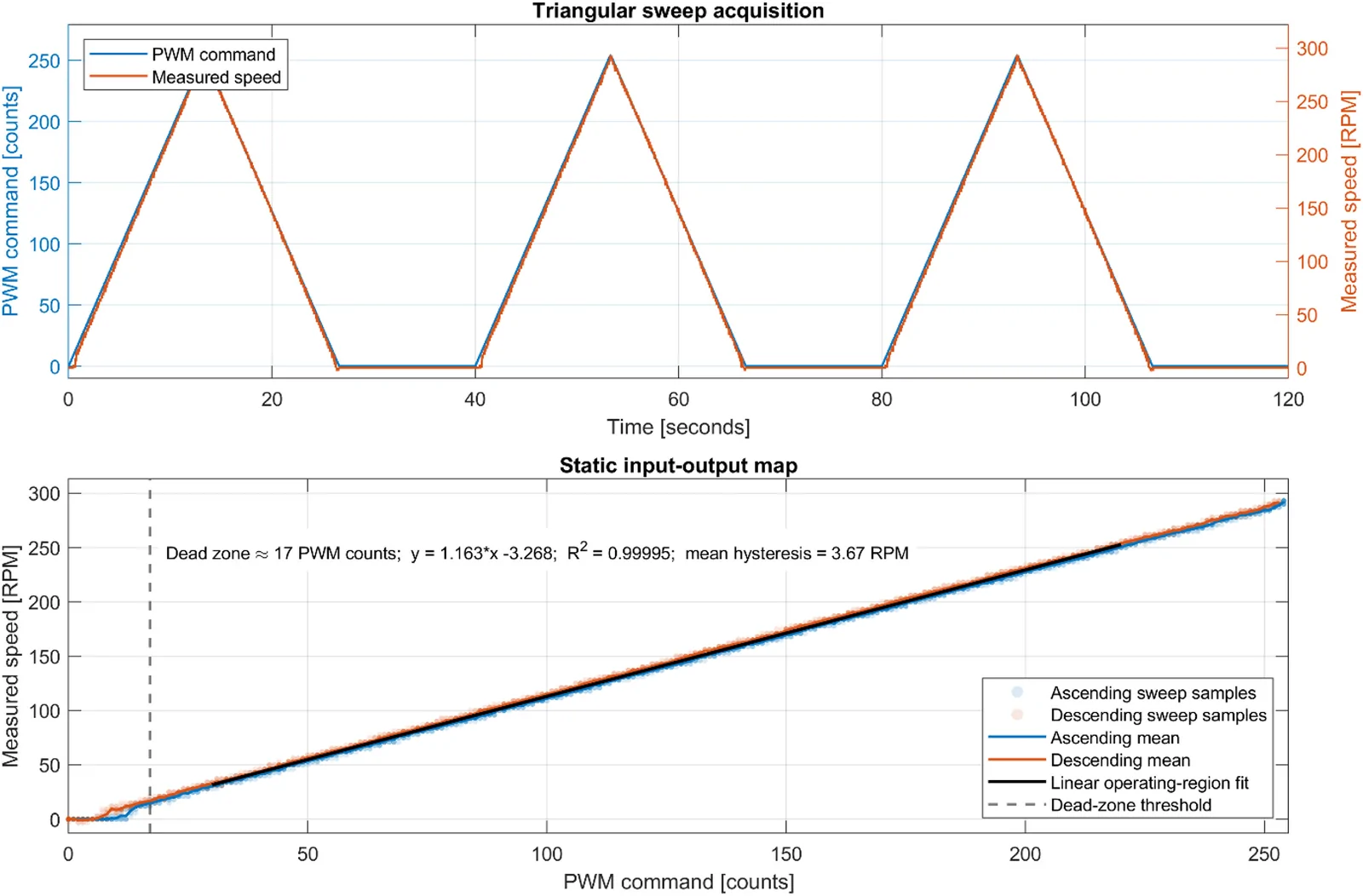
Static DC motor characterization from a triangular PWM sweep at a nominal 20 ms sampling time. Top: commanded PWM and measured speed during acquisition. Bottom: ascending and descending speed samples binned by PWM command, with branch means, dead-zone threshold, and linear operating-region fit.

For system identification, APRBS excitation logs were collected in system-identification mode at nominal sample times of 2 ms, 10 ms, 20 ms and 50 ms [7]. The GUI fit screenshots report continuous-time transfer function structures with zero-zero/one-pole, zero-zero/two-pole, and one-zero/two-pole forms. The MATLAB summary script reads each raw log, reconstructs the acquisition time vector, plots PWM input and speed output, stores raw-log summaries, and writes the transcribed GUI model coefficients and metrics to CSV. The APRBS input alternated between zero and an active integer PWM level from 80 to 240 counts, with each phase lasting 1250 ms. The realized excitation is retained in the raw logs. Model fitting in the GUI used SciPy least_squares with soft_l1 loss, f_scale = 0.5, x_scale = 'jac', and max_nfev = 3000. The fitting implementation is preserved in the archived GUI source. Fig. 3 shows the four APRBS acquisitions and the GUI RMSE values.

**Fig. 3:**
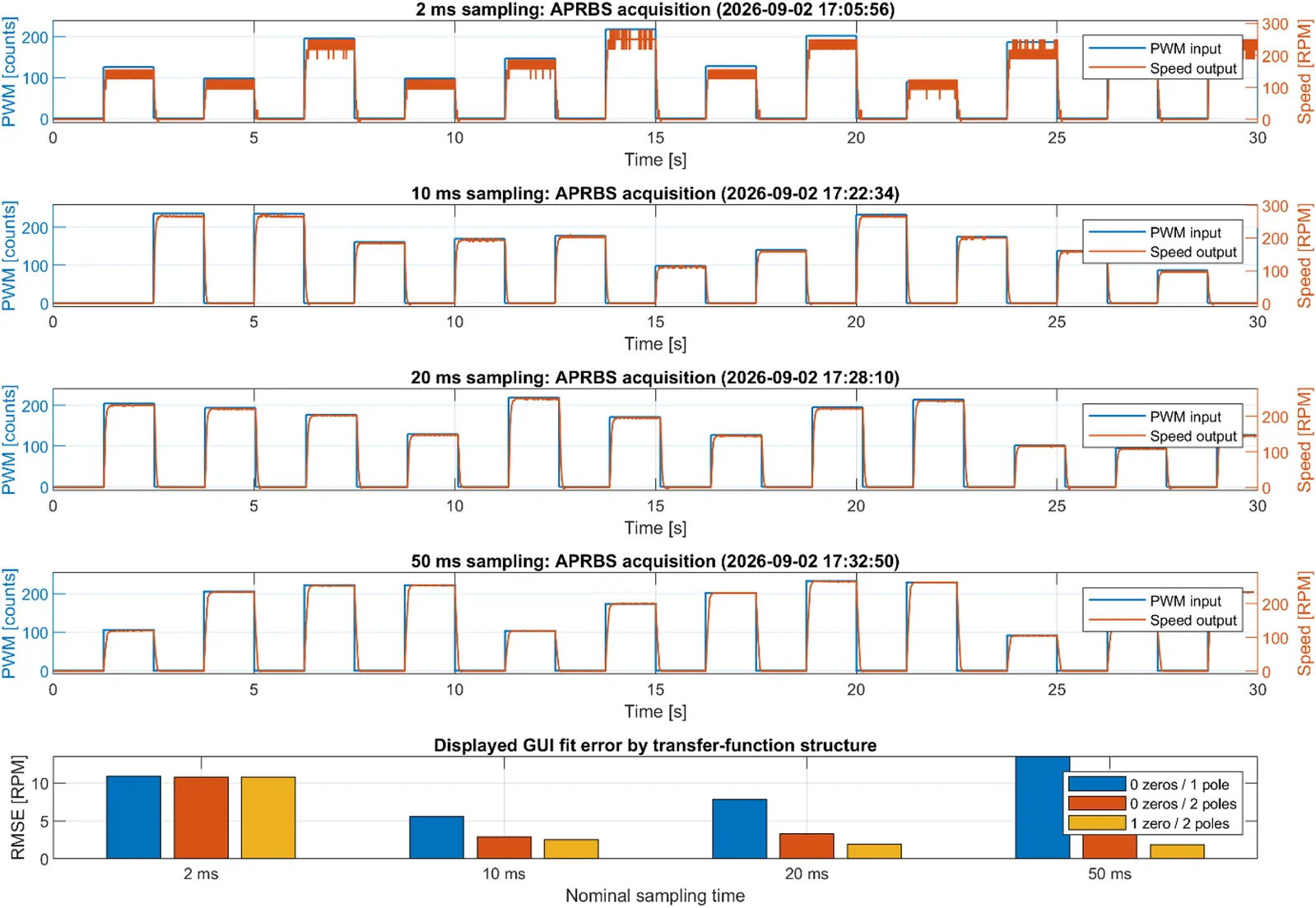
APRBS system-identification acquisitions and GUI-transcribed fit metrics. The upper panels show PWM input and measured speed at nominal sampling times of 2 ms, 10 ms, 20 ms and 50 ms; the lower panel summarizes displayed RMSE values for the tested transfer function structures.

The transfer function coefficients were used to simulate each model without refitting. The analysis used the last min(15,000, N) samples of each acquisition to reproduce the GUI data window, where N is the number of raw samples. Simulations used zero initial conditions and first-order-hold interpolation of the PWM input. Residuals were calculated as measured speed minus simulated speed, and RMSE was calculated as the square root of the mean squared residual.

The analysis also calculated residual autocorrelation at lags of 1–20 samples and input–residual cross-correlation at lags of 0–20 samples. The complete residual and correlation vectors are stored in system_identification_results.mat, while numerical summaries are provided in system_identification_model_results.csv.

Akaike Information Criterion (AIC) and Bayesian Information Criterion (BIC) were also calculated from the residual sum of squares and the number of estimated transfer function coefficients. In this application, these criteria provide a common numerical basis for comparing candidate model structures while accounting for both residual error and model parameter count. The parameter count excluded the normalized leading denominator coefficient and was set to 2, 3, and 4 for the zero-zero/one-pole, zero-zero/two-pole, and one-zero/two-pole structures, respectively. ΔAIC and ΔBIC were calculated for each sampling-time dataset by subtracting the minimum criterion value within the corresponding three-model group. These criteria were calculated from the residual sums of squares of the archived models after robust fitting. They describe performance on the identification records themselves, rather than on an independent validation dataset (Table 5).

**Table 5:** RMSE values were obtained from the GUI. ΔAIC and ΔBIC are referenced to the minimum AIC and BIC values, respectively, within each sampling-time group.

Ts (ms)	Model structure	RMSE (RPM)	ΔAIC	ΔBIC
2	0Z/1P	10.88	420.94	406.54
2	0Z/2P	10.73	6.7798	0
2	1Z/2P	10.73	0	0.83602
10	0Z/1P	5.6	4774.1	4762.1
10	0Z/2P	2.88	779.98	773.97
10	1Z/2P	2.53	0	0
20	0Z/1P	7.78	4213.6	4203
20	0Z/2P	3.29	1634.8	1629.5
20	1Z/2P	1.9	0	0
50	0Z/1P	13.5	2401.8	2393
50	0Z/2P	6.24	1480.6	1476.2
50	1Z/2P	1.81	0	0

For continuous controller validation, the GUI PID tuner was used to configure PI controllers, which were deployed through the firmware incremental implementation with trapezoidal integration. Validation logs were collected at controller loop periods of 5, 10, 20, and 50 ms using an automatic reference sequence with a 3000 ms period setting. Kd was zero in all four cases. Active reference levels ranged from 80 to 240 RPM and alternated with zero every 1500 ms. The approximately 0.3 s settling time shown by the tuner was a simulated design target. Fig. 4 shows the PID tuner for the 20 ms controller case. The folder-level MATLAB scripts call `validation_helpers/normalized_step_validation.m` to detect nonzero reference intervals, subtract local pre-step speed, normalize step responses, and export validation figures and result files. Table 6 lists the plant model and controller gains used in each acquisition. Fig. 5 shows the recorded signals and normalized responses for the continuous PI validation at a 20 ms loop period.

**Fig. 4:**
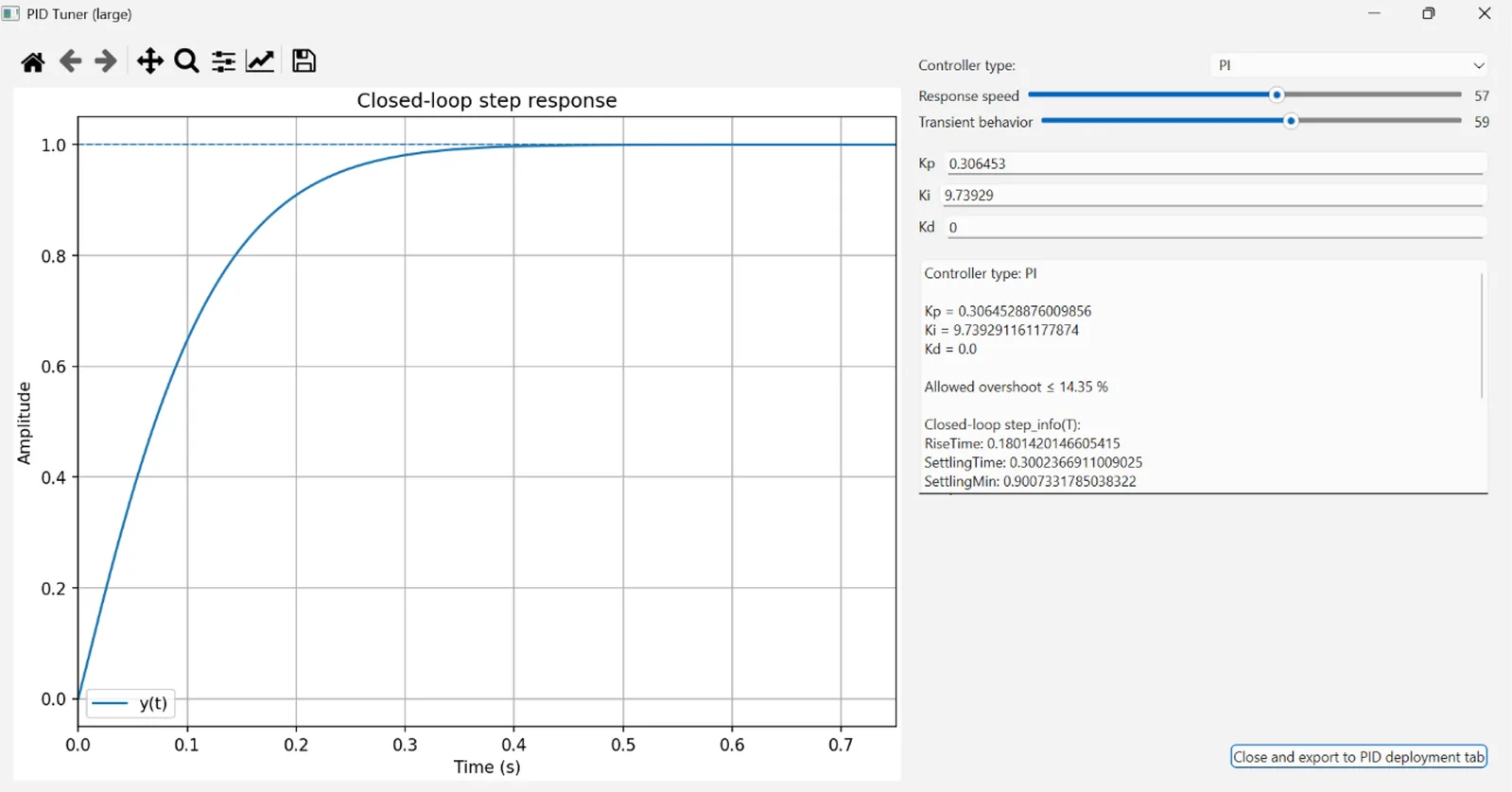
OpenMCT PID tuner screenshot for a continuous PI controller design. The interface displays the simulated closed-loop step response, selected controller type, tuned gains, and step-response metrics before firmware deployment.

**Table 6:** Identified plant models and continuous PI controller parameters used in the four experimental validation acquisitions. The plant-identification sampling time indicates the acquisition period of the dataset used to obtain each continuous-time plant model, whereas the controller loop time indicates the nominal firmware execution period used during controller validation.

Controller loop time (ms)	Plant ID sampling time (ms)	Identified plant G(s)	$K_{p}$	$K_{i}$
5	2	$\frac{41.77}{s+36.49}$	0.303452	11.3847
10	10	$\frac{32.22}{s+28.17}$	0.307647	10.5781
20	20	$\frac{25.59}{s+22.37}$	0.306453	9.73929
50	50	$\frac{15.38}{s+13.33}$	0.310369	7.95321

**Fig. 5:**
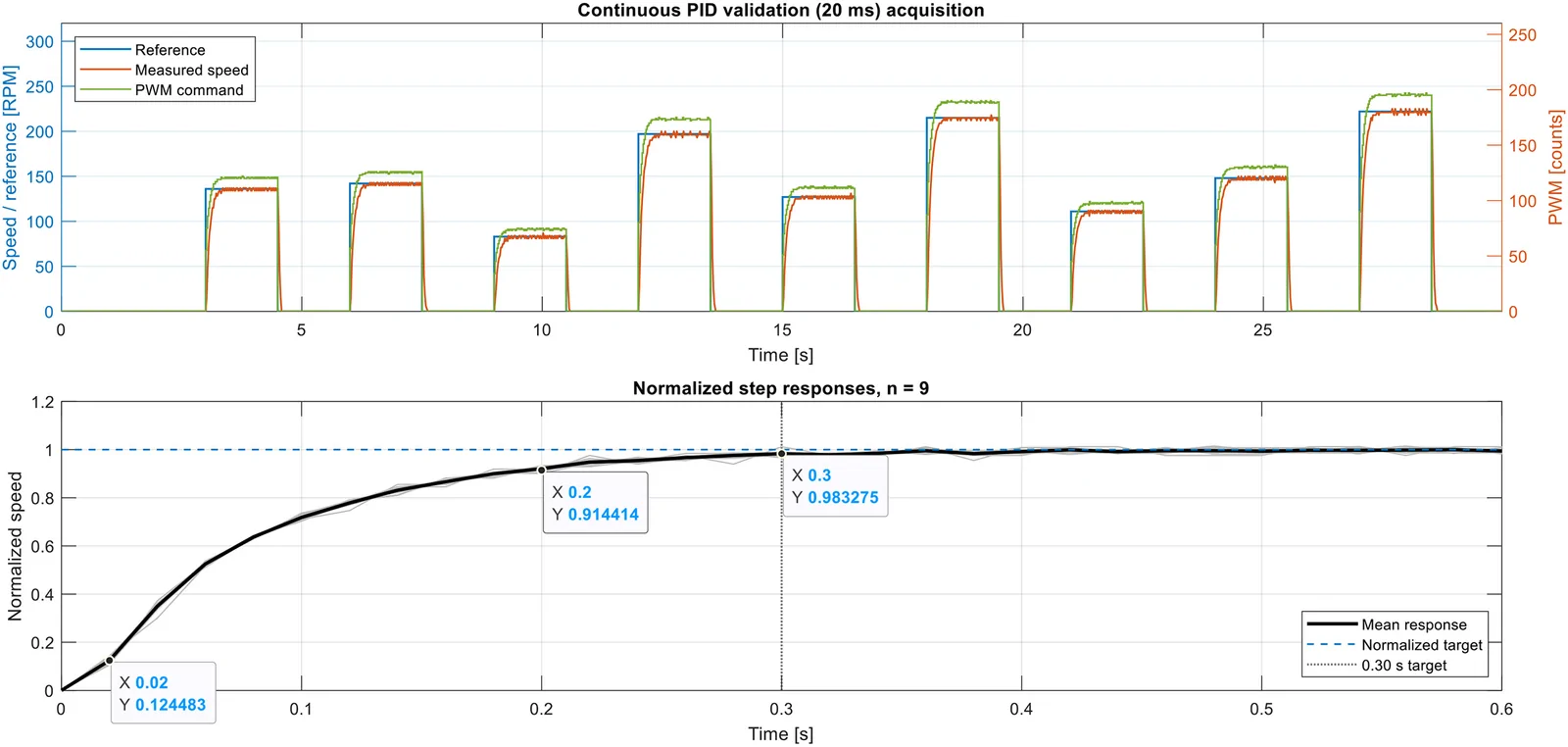
Continuous PI controller validation data for a representative 20 ms loop-time acquisition. Top: speed reference, measured speed, and PWM command recorded by the GUI. Bottom: normalized step responses, individual traces, mean response, and normalized target.

For both continuous and discrete controller validation, active intervals were identified by |REF| > 20 RPM. The baseline speed was the median measured speed during the preceding 0.20 s. Each response was normalized by subtracting this baseline and dividing by the difference between the active reference and the baseline. Valid responses were interpolated onto a common time grid, and their mean and sample standard deviation were calculated. No statistical outlier rejection was applied. The complete interval-selection and response-metric rules are documented in validation_helpers/README.md.

For discrete controller validation, three controller cases were implemented: an integrator with low gain, an integrator with higher gain, and an integrator with a real zero at 0.66. All three discrete-controller validation cases were designed using the same discrete plant at $T_{s}=0.02$ s. The discrete plant was obtained by zero-order-hold discretization of the identified continuous-time model G(s) = 25.59/(s + 22.37) at Ts = 0.02 s. The resulting GUI-transcribed model was G(z) = 0.41263z⁻¹/(1 − 0.63929z⁻¹). The discrete-controller folders include the raw speed-control log, GUI screenshots of the plant/controller design, MATLAB validation script, result file, and exported figures. Table 7 lists the controller parameters, while Fig. 6, Fig. 7 show the controller design and experimental validation for case B.

**Table 7:** Discrete controller parameters used in the three experimental validation cases at $T_{s}=20$ ms. C(z) denotes the base compensator before application of the selected root-locus gain K. The numerator and denominator vectors describe the complete implemented transfer function K·C(z), expressed in increasing powers of z⁻¹.

Case	Controller structure	Gain K	Base compensator C(z)	Implemented TF numerator b	Implemented TF denominator a
A	Integrator	3.86278	0.02/(z−1)	[0, 0.0772556, 0, 0]	[1, −1, 0, 0, 0]
B	Integrator	13	0.02/(z−1)	[0, 0.2600000, 0, 0]	[1, −1, 0, 0, 0]
C	Integrator + real zero at 0.66	9.9	(0.02z−0.0132)/(z−1)	[0.1980000, −0.1306800, 0, 0]	[1, −1, 0, 0, 0]

**Fig. 6:**
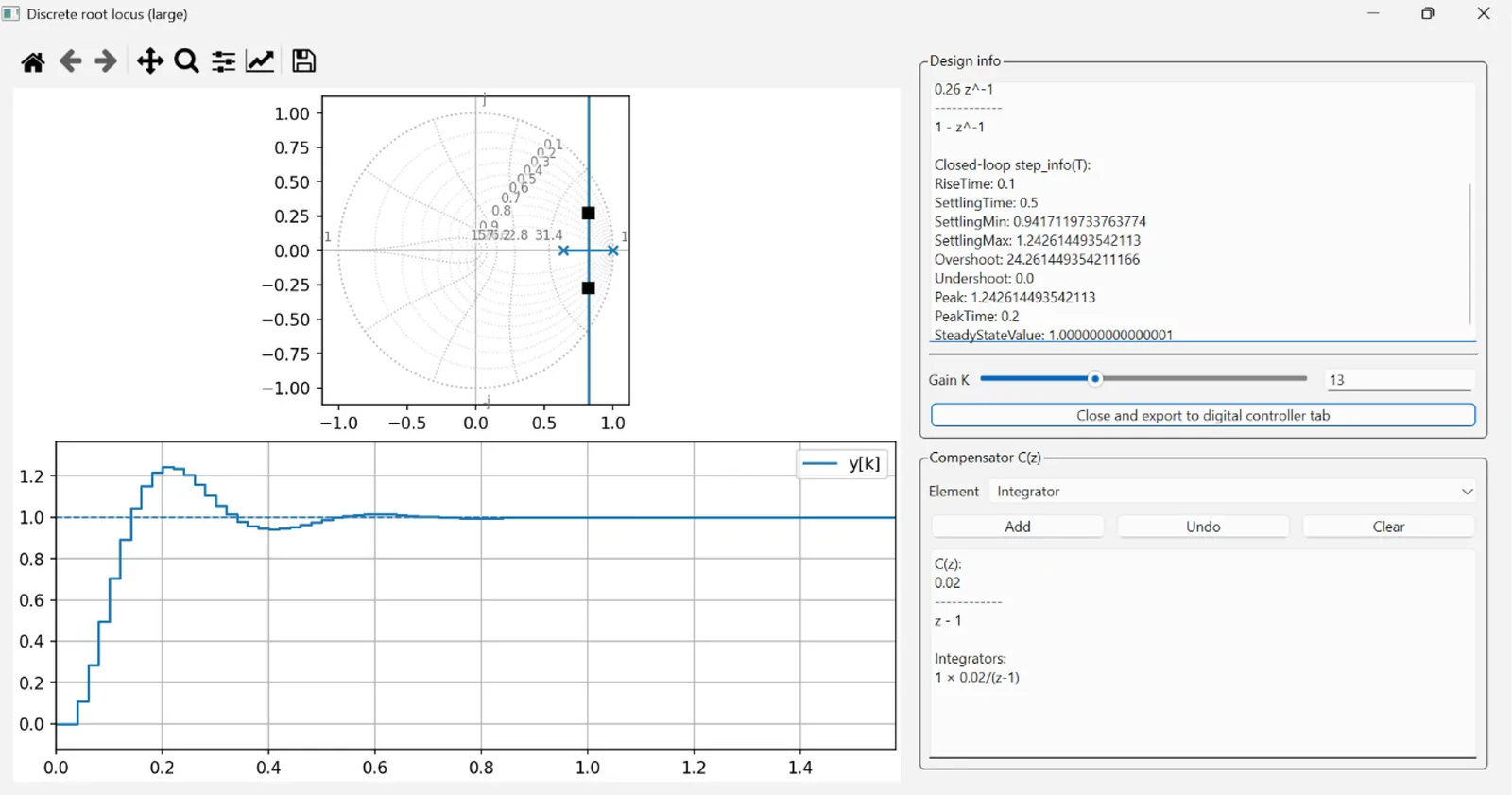
OpenMCT discrete-controller design screenshot. The GUI shows the discrete root locus, simulated step response, integrator compensator at Ts = 0.02 s, selected gain, controller expression, and displayed step-response metrics.

**Fig. 7:**
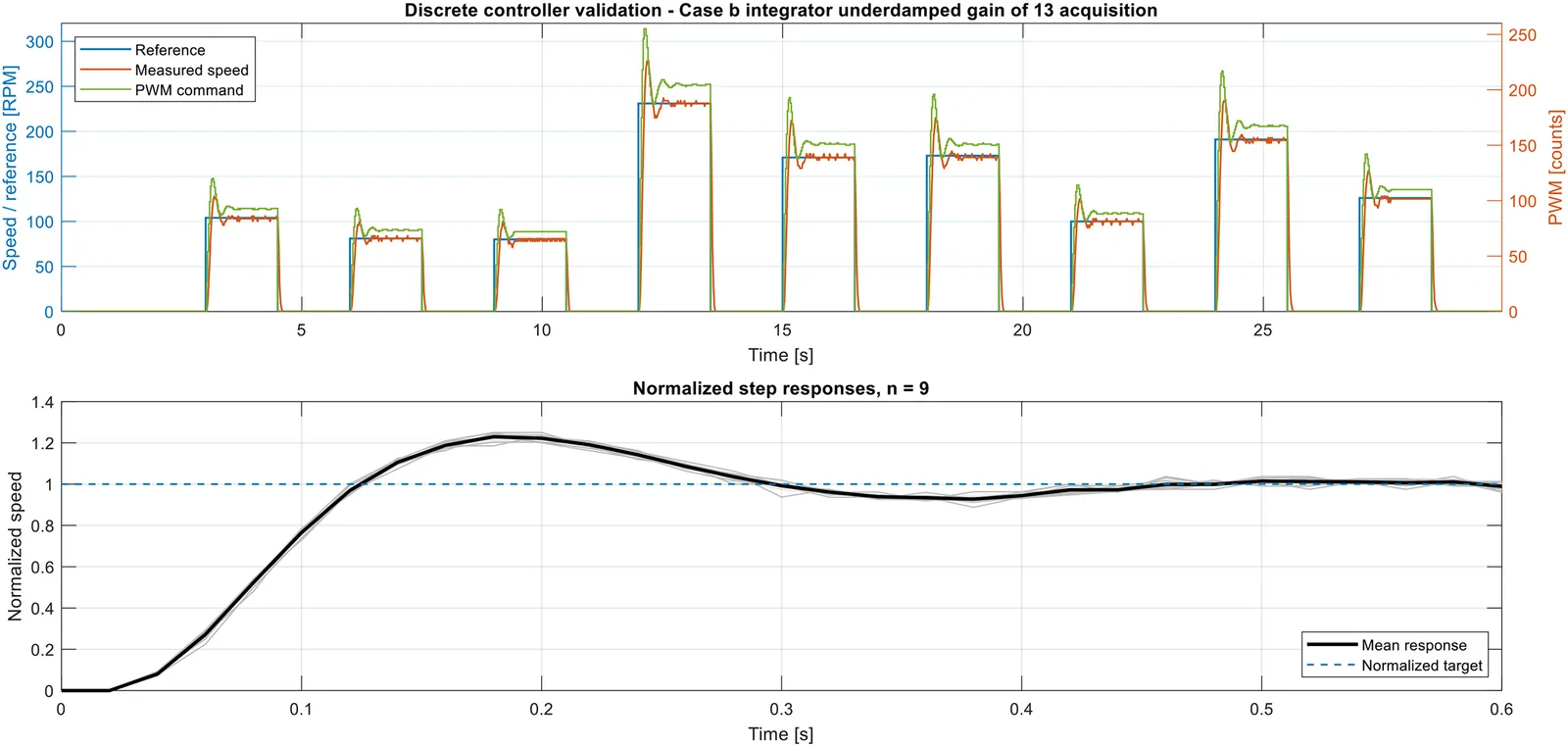
Discrete controller validation data for case B, integrator with gain K = 13 at Ts = 0.02 s. Top: recorded reference, measured speed, and PWM command. Bottom: normalized detected step responses and mean normalized response.

In the OpenMCT GUI implementation, the numerator is represented by [A B C D] and the denominator by [1 −E −F −G −H]. The denominator vectors in Table 7 therefore correspond to feedback coefficients [E F G H] = [1 0 0 0].

An additional chirp-excitation characterization run was recorded at a nominal 20 ms sample time. The MATLAB script reconstructs time from `DT_ms', plots the chirp command and measured speed response, and stores chirp metadata and DMM sample information when available.

As a first reuse exercise, extract the dataset and open 04_continuous_PID_validation/20_ms in MATLAB. The following commands reconstruct elapsed time and plot the reference and measured speed directly from the raw log. This example requires neither the acquisition hardware nor the OpenMCT GUI.

*T = readtable(`raw_data_20ms.txt',`NumHeaderLines',2);*
*time_s = [0; cumsum(T.DT_ms(1:end-1))]/1000;*
*plot(time_s,T.REF,time_s,T.MEAS);*
*xlabel(`Time (s)');*
*ylabel(`Speed (RPM)');*
*legend(`Reference',`Measured');*

Running PID_validation from the same folder reproduces the processed validation figure and MAT results. Students can compare the recorded response with the normalized responses generated by the script.

## Limitations

Digital multimeter current samples were acquired asynchronously with respect to the firmware loop, so the raw files include DMM timestamp, sample age, and sample identifier fields for synchronization between samples. The dataset does not include repeated trials across multiple motors or power supplies. DMM_AGE_ms describes the logged age of an asynchronous reading and does not account for the complete instrument integration and communication delay. The APRBS sequences and controller gains differ between acquisitions, so comparisons across sampling periods also reflect these differences.

A zero standard deviation in the stored DT_ms values does not establish the absence of timing variations below the recorded resolution. The chirp acquisition retains integer-millisecond intervals. Its nominal excitation frequency exceeds the 25 Hz Nyquist frequency after 125 s, so the final segment cannot be used as an unaliased frequency-response measurement.

## Ethics Statement

The authors have read and followed the ethical requirements for publication in Data in Brief and confirm that the current work does not involve human subjects, animal experiments, or any data collected from social media platforms.

## CRediT Author Statement

**Alejandro Von Chong:** Conceptualization, Methodology, Software, Validation, Writing – Original Draft. **Dorindo Cárdenas:** Writing- Reviewing and Editing.

## Declaration of generative AI and ai-assisted technologies in the manuscript preparation process

During the preparation of this work, the authors used ChatGPT (OpenAI) to assist with developing MATLAB scripts, reviewing the manuscript, and drafting README documentation for the experiments. The authors reviewed and edited the output as needed and take full responsibility for the content of the published article.
